# Establishment of a three-dimensional* in vitro *peri-implant bone-mucosa composite model

**DOI:** 10.1186/s12903-025-06930-2

**Published:** 2026-01-14

**Authors:** Behnaz Malekahmadi, Marjan Kheirmand-Parizi, Carina Mikolai, Andreas Winkel, Muhammad Imran Rahim, Katharina Doll-Nikutta, Andreas Kampmann, Nils-Claudius Gellrich, Dagmar Wirth, Henning Menzel, Meike Stiesch

**Affiliations:** 1 Department of Prosthetic Dentistry and Biomedical Materials Science, Hannover Medical School, Carl-Neuberg-Str. 1, Hannover, 30625 Germany; 2 Lower Saxony Centre for Biomedical Engineering, Implant Research and Development (NIFE), Stadtfelddamm 34, Hannover, 30625 Germany; 3 Department of Oral and Maxillofacial Surgery, Hannover Medical School, Carl-Neuberg-Strasse 1, Hannover, 30625 Germany; 4 Model Systems for Infection and Immunity, Helmholtz Centre for Infection Research, Inhoffenstraße 7, Braunschweig, 38124 Germany; 5 Institute of Experimental Medicine, Hannover Medical School, Carl-Neuberg-Str. 1, Hannover, 30625 Germany; 6 Institute for Technical Chemistry, Braunschweig , University of Technology, Hagenring 30, Braunschweig, 38106 Germany

**Keywords:** Bone model, Oral mucosa, Hard tissue, Soft tissue, 3D *in vitro* model, Organotypic model, Peri-implantitis

## Abstract

**Background:**

Peri-implant health depends on the complex interactions between the dental implant, surrounding soft/hard tissues and the oral microbial environment. However, existing 2D and monoculture models fail to replicate this complexity, limiting their clinical relevance. Therefore, this study aimed to develop a clinically relevant 3D in vitro model that integrates oral soft tissue, hard tissue and a titanium implant in a 3D setup to accurately replicate the peri-implant environment. In addition, the model was designed to integrate bacterial biofilms, in order to mimic incipient peri-implant infections.

**Methods:**

As a hard tissue component, osteoblast-covered HA/TCP scaffold structures were developed and merged with peri-implant mucosa, resulting in a 3D in vitro peri-implant bone-mucosa composite model. The composite model was then cultivated for 2, 7 and 14 days. At each time point, histological analysis, live/dead staining and collagen immunofluorescence staining were performed to assess its structural integrity, osteoblast viability and bone ECM characteristics. To demonstrate proof-of-concept for suitability in simulating implant infection, an oral multispecies biofilm was integrated on top of the implant in the peri-implant bone-mucosa model.

**Results:**

Cell viability and osteoblastic phenotype were maintained throughout the study period. Microscopic and histological analyses confirmed a homogenous structure, with a stratified epithelium overlying collagen-embedded human gingival fibroblasts closely connected to the underlying scaffold structure interspersed with bone cells. Combined with a living multispecies biofilm, this model represents several essential components observed in peri-implant interaction.

**Conclusions:**

By combining oral soft tissue, hard tissue and a titanium implant in a 3D setup, this model represents the first and most complex model for evaluating innovative implant materials and novel treatment strategies as well as studying the development of peri-implant diseases. Incorporating different biofilms could enhance the model’s clinical relevance, enabling the study of pro-inflammatory responses to bacterial infections in a setting that includes both soft and hard tissue.

**Supplementary Information:**

The online version contains supplementary material available at 10.1186/s12903-025-06930-2.

## Background

Dental implants are widely used to replace missing teeth, offering a durable solution that restores both function and aesthetics of the natural tooth [1]. The success and longevity of dental implants depend on several factors, including complete osseointegration, the formation of a protective soft tissue seal and the absence of infection [2–5]. Infections trigger inflammation, which leads to tissue destruction of soft tissue as observed for peri-implant mucositis and additional bone loss in case of peri-implantitis, which in turn facilitates the further penetration of bacteria into the tissue [1, 6]. Therefore, ensuring a sufficient soft tissue seal around implants represents an essential part in protecting the underlying tissues and preventing bacterial infections [3]. Considering the complex interactions among soft tissue, hard tissue, implant material and bacteria is crucial not only for studying peri-implant disease development but also when evaluating innovative implant modifications and novel therapeutic strategies [7, 8].

The evaluation of such strategies for implant dentistry in order to reduce implant-related complications has traditionally relied on either two-dimensional (2D) in vitro models or animal models [9–11]. Although 2D models are reproducible, cost-effective, and suitable for assessing individual parameters, they fail to reflect the complexity of clinical situations, often leading to cellular responses that deviate from observations in vivo [12–14]. Cells grown in 3D, like in natural tissues or cells cultivated in monolayers, like on cell culture plastic, exhibit differences that are well described. For instance, osteoblasts can undergo changes in their gene expression and cytoskeleton structure as a result of different physical environments [14, 15]. Additionally, monoculture models often overlook the complex interactions between various cell types, limiting their relevance to complex clinical settings [9, 16]. Preclinical animal models are well established to address these limitations, but they raise ethical concerns, demand considerable time and resources and present interspecies differences in molecular and physiological conditions that may mislead interpretation of the results [17]. Recent advances and mandatory considerations of 3R principles (replacement, reduction and refinement) have promoted the development of alternative physiologically relevant 3D in vitro models to better understand the complex interactions between different cell types with modified implant surfaces and to translate new findings into in vivo applications more effectively [10, 18]. These 3D in vitro models can more accurately replicate the native tissue’s structural and functional features by improving cell-to-cell and cell-to-matrix interactions, enhanced nutrient and oxygen diffusion, mimicking tissue architecture and supporting a more accurate physiological environment compared to traditional 2D cultures [17, 19].

Recently, 3D in vitro models have been created to mimic physiological processes involved in dental implant treatment [20–22]. These 3D models were developed to investigate either soft tissue-implant interaction or hard tissue-implant interactions [21]. Some studies have also incorporated bacteria to assess the interactions between the implant, soft tissue, and bacterial infection [20, 22–24]. However, so far no study has combined all these factors—soft tissue, hard tissue and implant— nor has any incorporated all of these together with bacteria to closely resemble the clinical situation. Moreover, an accurate evaluation especially of processes connected to peri-implantitis should require the inclusion of bone tissue in implant models. Therefore, the aim of this study was to develop a 3D in vitro peri-implant bone-mucosa model serving as platform for future investigations to assess the interactions of modified implant surfaces/biomaterials within both soft and hard tissue. Furthermore, by co-culturing the model with bacterial biofilms, the development and treatment of peri-implant diseases in a clinically relevant setting can be studied. For this purpose, in a first step a 3D in vitro bone-implant model was established and quantitatively and qualitatively analyzed to assess bone-like tissue formation. This scaffold-based hard tissue was then merged with our previously developed oral mucosa model [25], creating a comprehensive 3D peri-implant bone-mucosa system. Microscopic and histological evaluations were performed to assess its ability to replicate native tissue structures in terms of phenotypic characteristics, histology and expression of osteoblastic phenotype. By combining both bone and mucosal tissues, this model is the first to successfully integrate both environments surrounding a dental implant. Furthermore, this model was exemplarily used to integrate an oral multispecies biofilm (MSBF), creating a complex 3D co-culture system including soft and hard tissue as well as biofilm and implant, which can be used in future studies of infections in a setting similar to the clinical situation.

## Methods

### Cell culture, media and reagents

Normal Human Osteoblasts (NHOst, CC-2538, Lonza) were cultured in Alpha Minimum Essential Medium (αMEM, P04-21250, Lonza) supplemented with 12% fetal bovine serum (FBS, P30-3306, PAN-Biotech GmbH) and 1% penicillin/streptomycin (P/S, P0781, Sigma-Aldrich). Human gingival fibroblasts (HGFs, 1210412, Provitro GmbH) were cultured in Dulbecco’s Modified Eagle’s Medium (DMEM, P04-04500, PAN-Biotech GmbH) with 10% FBS and 1% P/S. Keratinocyte Serum-Free Medium (KerSFM, 10725-018, Gibco Life Technologies) supplemented with 0.2 ng/ml human recombinant Epithelial Growth Factor (EGF, 10450-013, Gibco Life Technologies), 25 µg/ml Bovine Pituitary Extract (BPE, 13028-014, Gibco Life Technologies), 0.3 mM calcium chloride (CaCl2, C-34006, PromoCell) and 1% P/S was used to cultivate immortalized Human Oral Keratinocytes (OKF6/TERT-2) [26]. All three cell types were grown under a humidified environment at 37 °C with 5% CO_2_. Once the cells reached 70–80% confluence, NHOst cells were detached using Accutase^®^ solution (A6964, Sigma-Aldrich), while HGF and OKF6 cells were detached with trypsin/EDTA (P10-020100, PAN Biotech). In all experiments, NHOst cells were used at passages 6–8, HGF cells at passage 8–10 and the OKF6 cell Line at passages 25–35.

### Scaffold selection

Three different types of scaffolds with the following features (Table 1) were purchased commercially and used initially without any modifications. Scaffolds were examined and compared for cell seeding efficiency (CSE), cell viability and cell growth. In 24-well-plates different numbers of NHOst (PS: 2.12 × 10^5^ cells, PCL: 3.92 × 10^5^ cells, HA/TCP: 1 × 10^6^ cells) were statically seeded on the top of the scaffolds by dropping (five separate drops). Cell seeding densities on PCL and PS scaffolds were selected according to the manufacturer’s protocol, based on the 3D growth surface area. The CSE was calculated after 24 h of incubation according to the formula: CSE (%) = 1- ((cells left in the well + non adherent cells)/cells seeded on scaffold) × 100. Live/dead staining of cells on scaffolds was performed one day after seeding. Osteoinductive medium containing αMEM medium supplemented with 12% FCS, 1% P/S, 0.1 mM Ascorbate (Sigma-Aldrich, Merck KGaA), 5 mM β-Glycerophosphat (Sigma-Aldrich), 10 nM Dexamethason (Sigma-Aldrich) was added at day 3 and was refreshed every 2–3 days in the following 14 days of cultivation to evaluate mineralization capacity of NHOst on PS and PCL scaffolds. Detailed explanations of analytical methods are provided in the following sections.

**Table 1:** General characteristics of scaffolds

Type of scaffold	Material	Size	Company
3D Biotek 3D Insert™ PS scaffold	Polystyrene	24-well compatible, Thickness 0.6 mm	PS152024-12, 3D-Biotek, LLC.-, New Jersey, USA
3D Biotek PCL scaffold inserts	Polycaprolactone	24-well compatible, Thickness 1.6 mm,	PCL303024-BR, 3D-Biotek, LLC.-, New Jersey, USA
ReproBone discs	60% Hydroxyapatite, 40% β-Tricalcium phosphate	10 mm diameter, 5/2 mm height	10RB10D2, Ceramisys, Ltd., Sheffield, UK

### Scaffold preparation

Based on the findings in the initial experiments, ReproBone discs (synthetic resorbable bone graft substitute, HA/TCP) were selected for the further development of a 3D bone-implant model. The size of scaffolds was customly adjusted by the company to 10 mm in diameter and 2 mm in height. In order to increase nutrient supply within scaffolds as well as preparing the implant insertion site, a perpendicular hole in the center of each scaffold was drilled using a 2.5 mm round end taper dental bar (ZR6856.314, Komet Dental. Gebr. Brasseler GmbH) with 200,000 rpm/min speed. To increase the precision of this preparation, a drill guide was produced in-house. Additionally, 5–6 small perforations were created in the remaining scaffold ring with insulin syringes 0.5 ml (0.30 mm × 8 mm, BD Micro-Fine, Becton, Dickinson and company) to further improve medium circulation and cell distribution. Loose fragments were removed by washing scaffolds twice with cell culture medium. Each side of scaffolds was sterilized using UV light (UV-C disinfection box, Philips, 135 W) for 15 min.

### Assembly of a 3D in vitro bone-implant model

Scaffolds were soaked in αMEM medium for 24 h prior to seeding. Afterwards, scaffolds were placed into 12 well plates with the bottom covered by a layer of parafilm. A density of 0.8–1.5 × 10^6^ cells/scaffold in 30 µl αMEM medium was seeded on each scaffold (15 µl each top and bottom). The cells were allowed to adhere at 37 °C in 5% CO_2_ while avoiding plates’ agitation. After two hours, the scaffolds were transferred into a 6 well plate, submerged with 4 ml αMEM medium and cultured overnight under static conditions. After 24 h, scaffolds were transferred into cell culture inserts (0.45 μm Millicell, 30 mm diameter, Merck Milipore Ltd), which were primarily perforated. The inserts were placed in 6-well plates and filled with medium in- and outside to cover the scaffolds. After two days, the medium was changed to osteoinductive medium, changing it every 2–3 days. After 14 days, scaffolds were placed into a 6 well plate and a sterile titanium cylinder (machined surface, grade 4, 3 mm diameter, 2.3 mm height) was gently inserted. These constructs were further incubated for 48 h under static culture conditions in 4 ml of osteogenic medium.

### Assembly of a 3D in vitro peri-implant bone-mucosa composite model

The assembly of the peri-implant mucosa model followed the previously established protocol [25]. Briefly, HGFs (4 × 10⁵ cells/model) (121 0412, Provitro GmbH) were embedded in a collagen type-I hydrogel mix (2 mg/mL bovine collagen type-I (PureCol^®^, 5005-100ML, Advance Biomatrix) supplemented with FBS, L-glutamine (G7513, Sigma-Aldrich), 10 x DMEM (P03-01510, Pan-Biotech) and reconstitution buffer (2 mg/mL sodium bicarbonate, 2 mM HEPES and 0.0062 N NaOH)). After 4 days of cultivation, a titanium cylinder (machined surface, grade 4, 3 mm in diameter, 4.3 mm in height) pre-colonized in upper section with HGFs was integrated into the HGF-hydrogel. For this purpose, the HGF-hydrogel and the membrane of the culture insert below were punched using a 2.5 mm diameter biopsy punch. The titanium cylinder was inserted in the HGF-hydrogel and through the membrane of the culture insert, with approximately 2 mm of its height (non-pre-colonized part) extending below the insert. The remaining procedure followed the previously described protocol, with OKF6 cells seeded on top of the gel. The tissues were subsequently placed at an air-liquid interface and cultivated with airlift medium (3:1 DMEM (P04-03591, Pan-Biotech) and Ham’s F-12 (P04-14559, Pan-Biotech), supplemented with 5 µg/mL insulin, 0.4 µg/mL hydrocortisone, 2 × 10^−11^ M 5-triiodo-L-thyronine, 8 × 10^−5^ M adenine, 5 µg/mL transferrin, 10^−10^ M cholera toxin, 2 mM L-glutamine, 10% FBS, 1% P/S) for an additional two weeks to stimulate the epithelial differentiation and stratification. In parallel, the 3D bone model was constructed and cultivated for 23 days as described in assembly of bone model section, except without implant insertion.

On day 24, after completing the development of both models (3D peri-implant mucosa model, 3D bone model) separately, they were merged to create the 3D peri-implant bone-mucosa composite model. For this purpose, the soft tissues were carefully loosened from the inserts using a pipette tip to preserve their integrity and structure. Next, the culture insert’s porous membrane was precisely cut using a scalpel (Scalpel 21, Feather). Simultaneously, as the membrane was removed, the mucosa surrounding the implant was gently positioned onto the matured bone model, ensuring that the titanium Lined up with the central hole of the scaffold creating a 3D peri-implant bone-mucosa model. After merging, the models were individually placed in 6-well plates with 1 ml of airlift medium in each well. These models were then maintained under static cultivation for 2, 7 and 14 days, with the medium refreshed every 2 to 3 days.

### Assembly of a 3D in vitro peri-implant bone-mucosa-biofilm composite model

An oral multispecies biofilm (MSBF) consisting of *Streptococcus oralis* (ATCC^®^ 9811^TM^, American Type Culture Collection ATCC), *Actinomyces naeslundii* (DSM 43013, German Collection of Microorganisms and Cell Cultures), *Veillonella dispar* (DSM 20735) and *Porphyromonas gingivalis* (DSM 20709) was integrated into the 3D peri-implant bone-mucosa model as previously described [27]. Briefly, the four bacterial species were pre-cultured at 37 °C under anaerobic conditions (80% N₂; 10% H₂; 10% CO₂) in brain heart infusion (BHI) medium (CM1135B, Oxoid), supplemented with 10 µg/mL vitamin K. The bacterial pre-cultures were mixed equally in BHI/vitamin K to achieve a final optical density (600 nm) of 0.01 for each species. The MSBFs were cultivated on glass cover slips (18 mm in diameter, 1 mm in thickness, Thermo Scientific Menzel) in 12-well plates for 24 h under anaerobic conditions (less than 0.1% O_2_, 7–15% CO_2_) at 37 °C.

After the assembly of the 3D peri-implant bone-mucosa model and cultivation for 14 days, MSBF was placed with biofilm side on spacers and on the integrated titanium cylinder of the peri-implant bone-mucosa model, as previously described [20]. The co-culture model was then submerged cultivated in co-culture medium (airlift medium without P/S and supplemented with 10% BHI/vitamin K) for 24 h under a humidified environment at 37 °C with 5% CO_2_.

### Live/dead fluorescence staining and microscopy

Cell adhesion and viability on scaffolds were assessed after 1 day of cell seeding using a live/dead fluorescence staining. A similar evaluation was conducted after 17 days of cultivation in bone-implant model and also 2, 7 and 14 days after constructing the 3D peri-implant bone-mucosa model. A mixture of propidium iodide (P4864, Sigma-Aldrich) and Calcein AM (C3099, Thermo Fisher Scientific Inc.), each diluted 1:1000 in sterile PBS, was used to stain the cells in the scaffolds. The medium from each well was collected, replaced with 4 mL of the live/dead staining solution and further incubated for 30 min at 37 °C in a humidified environment with 5% CO_2_. All the staining procedure was protected from light. Subsequently, the staining solution was replaced with PBS to enable microscopic examination using Confocal Laser Scanning Microscopy (CLSM; Leica TCS SP8, Leica Microsystems). Images were taken using lasers with 488 nm and 552 nm wavelength. Reflection mode (405 nm) was used to observe scaffolds’ surface. After examination of cells on the scaffolds’ top surface in the bone-implant model, scaffolds were perpendicularly cut using disposable scalpels (No.11, Feather safety razor Co., Osaka, Japan) and turned 90° to visualize cell viability inside the scaffolds. The same cutting procedure was applied to the peri-implant bone-mucosa model. Three-dimensional image reconstruction was done using the Imaris x64 8.4 software package (Bitplane AG).

### Collagen 1 immunofluorescent staining and microscopy

At the endpoint of the bone-implant model (17 days) and after 2, 7 and 14 days of cultivation in the peri-implant bone-mucosa model, samples were fixed using 4% paraformaldehyde (PFA, 0335.2, Carl Roth GmbH) for 20 min at room temperature. Samples were permeabilized with 0.1% Triton X-100 (T9284, Sigma-Aldrich) in phosphate-buffered saline (PBS, D8537, Sigma-Aldrich) for 10 min at room temperature. After rinsing, the samples were blocked with 2% bovine serum albumin (BSA, A9418, Sigma-Aldrich) in PBS for 30 min at 37 °C to prevent nonspecific binding. Scaffolds were further incubated with the Collagen Type I Polyclonal primary antibody (Col-I, 1:2000, 14695-1-AP, Proteintech) for 2 h at 37 °C or overnight at 4 °C. After washing four times with PBS, the samples were incubated for 1 h at room temperature or 30 min at 37 °C with the secondary antibody conjugated to DyLight^®^ 488 (Goat Anti-Rabbit IgG H&L, 1:200, ab96883, Abcam). After washing four more times, Phalloidin–TRITC (1:500, P1951, Sigma-Aldrich) was used to visualize filamentous actin (F-actin). This counterstaining step lasted for 30 min at room temperature. The samples were again washed four times with PBS and CLSM was performed immediately (lasers with 405 nm, 488 nm and 552 nm wavelengths). Cell observation on the top and middle of the bone-implant model, as well as the middle of the bone part in peri-implant bone-mucosa model, was performed as described in “Live/dead fluorescence staining and microscopy” section. The Imaris software was used for 3D reconstruction of stained specimens.

### Alizarin Red S Staining (ARS) and quantification

After 17 days of cultivation, 3D bone-implant models were fixed using 4% paraformaldehyde for a duration of 20 min at room temperature while shaking on an orbital shaker (Titramax 100, Heidolph GmbH). The bone models were immersed with 2% Alizarin Red Staining solution (ARS, A5533, Sigma-Aldrich) for 30 min at room temperature in the dark while shaking. To measure the extent of mineralization, the ARS dye was extracted from the samples by submerging them in a mixture of 10% acetic acid (Carl Roth GmbH) and 20% methanol (JT Baker) at room temperature for 30 min while shaking. Subsequently, the absorbance was determined at 405 nm wavelength using a plate reader (Tecan, Infinite M200Pro). To ensure the accuracy of the matrix mineralization analysis, a control group of unseeded scaffolds was included, which was incubated in osteogenic medium in parallel with the experimental samples.

### Histological examination

The samples were fixed for at least 24 h in 4% buffered formalin solution at room temperature. Samples were dehydrated using an ethanol gradient (50%, 70%, 96%) and then infiltrated with Technovit 9100 resin solution (Kulzer GmbH). Polymerization of the samples was performed using fresh Technovit 9100 resin in embedding molds. Sectioning, grinding and Elastica Van Gieson staining were performed at either MORPHISTO GmbH (Offenbach am Main) or LLS ROWIAK LaserLabSolutions GmbH (Hannover). Microscopic evaluation was done using Zeiss Axioskop 40 microscope (Carl Zeiss GmbH).

### Microscopic visualization of MSBF

MSBFs were fluorescently stained with SYTO^®^9 and propidium iodide using LIVE/DEAD^®^ BacLight™ Bacterial Viability Kit (L7012, Thermo Fisher Scientific GmbH). A 1:1000 dilution of each stain was prepared in PBS and administered to the samples for 30 min of incubation in the absence of light. Subsequently, fixation of stained samples was applied using 2.5% glutaraldehyde solution (111-30-8, Carl Roth GmbH; diluted 1:10 with PBS). Biofilms were visualized utilizing a CLSM microscope with lasers at 488 nm and 552 nm wavelengths. The 3D reconstructions of stained MSBF were prepared using Imaris software.

### Statistical analysis

Statistical analyses and graphic processing of the data were performed using the GraphPad Prism Software 8.4. Normal distribution was checked using the Kolmogorov–Smirnov test. According to the results, Mann-Whitney test was used to analyze ARS staining results. A significance level of α = 0.05 was set for all comparisons.

## Results

### Scaffold selection and further adjustments

**Fig. 1:**
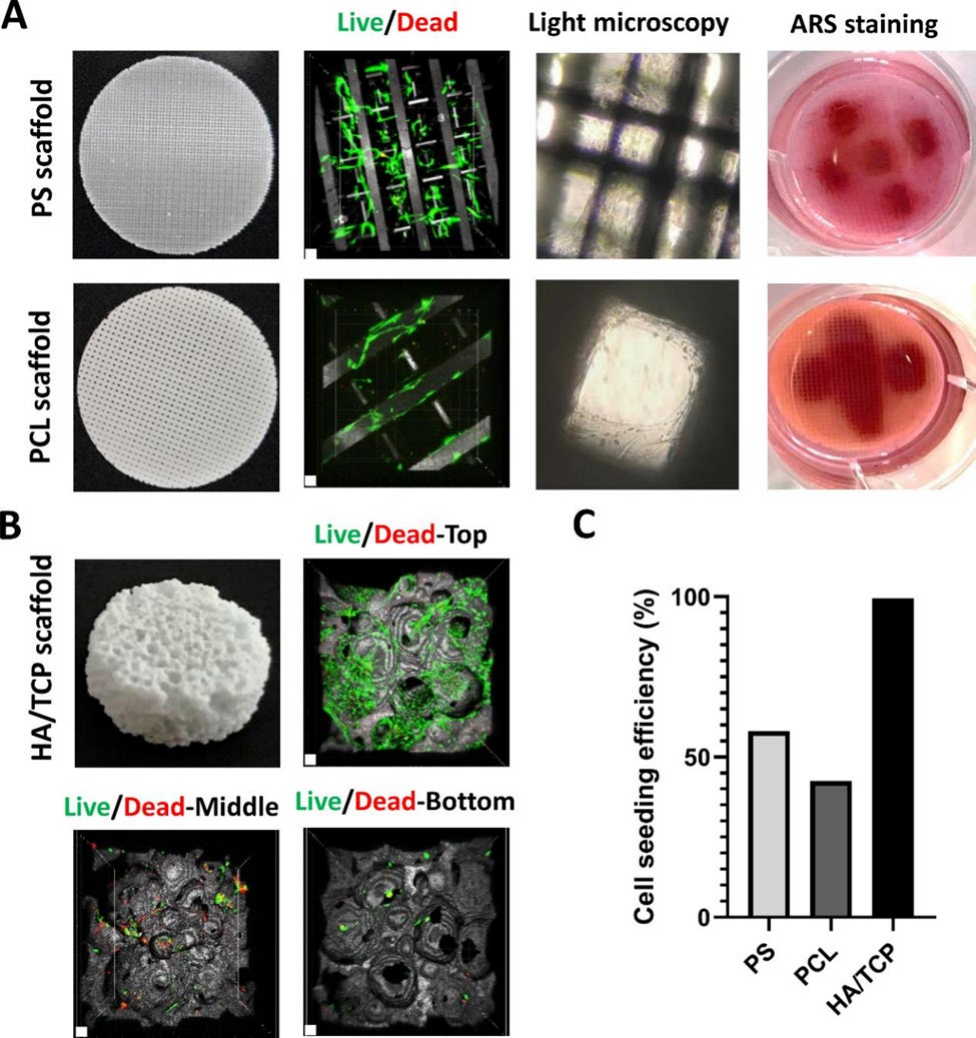
Biological evaluation and comparison of PS, PCL and HA/TCP scaffolds. (**A**) Cell viability, growth and mineralization on thin PS and PCL scaffolds. Live/dead images of NHOst cells on scaffolds were taken at day 1 after seeding. Light microscopy images (25x) and photographs of ARS staining were taken after 17 days of osteogenic cultivation. Scale bars represent 100 μm. (**B**) Photograph of unmodified thick HA/TCP scaffold (10 × 5 mm) and live/dead fluorescent images showing cell viability and penetration level one day after cell seeding. Images were taken from top, bottom and the middle by cutting the scaffolds perpendicularly. Scale bars represent 100 μm. (**C**) Comparison of cell seeding efficiency (CSE) between scaffolds one day after cell seeding. Data shown are representative of *n* = 2 independent experiments

Initially, a comparative analysis of three commercially available scaffolds with different chemical and physical characteristics (PS, PCL, HA/TCP, Table 1) was performed in order to identify the optimal scaffold that aligned with our objectives. We observed that cells seeded statically in drops on PS and PCL scaffolds attach and remain mainly at application site without spreading even after growth for longer cultivation periods. Accordingly, ARS staining visualizes a drop-related pattern of mineralization on PS and PCL scaffolds (Fig. 1A). While cell distribution and growth were limited in PS and PCL scaffolds, HA/TCP scaffolds exhibited further distribution of cells on the scaffold surface and in open cavities (Fig. 1B). Despite these differences, all three scaffolds demonstrated good cell viability in live/dead fluorescence staining (Fig. 1A, B). However, due to the thin structure of PS and PCL scaffolds, CLSM imaging could effectively capture cell viability across the depth, whereas for HA/TCP scaffolds, additional imaging from the middle and bottom was required to assess cell viability and penetration. Cell penetration within the HA/TCP scaffold in the dimension of 10 × 5 mm was limited in this initial analysis, resulting in minimal cell presence within the middle and bottom of the scaffold (Fig. 1B). Nevertheless, HA/TCP scaffolds displayed with 99.4% a much better seeding efficiency when compared to PS and PCL scaffolds with a CSE of 58% and 42.5%, respectively (Fig. 1C). Based on the more uniform distribution and increased CSE as well as a high compliance with actual bone material regarding chemical composition and mechanical characteristics, we decided to proceed with HA/TCP scaffolds to develop a 3D in vitro bone-implant model with enhanced clinical relevance. Although, a customized reduction of the HA/TCP scaffold size to 10 × 2 mm improved cell distribution and penetration, this approach did not support long-term cell maintenance, as 17 days of static culture still led to reduced cell viability in both the center and surface of the scaffold (Fig. S1). We hypothesized that cell viability was compromised by lack of nutrients, specifically in the inner compartment of the scaffold.

**Fig. 2:**
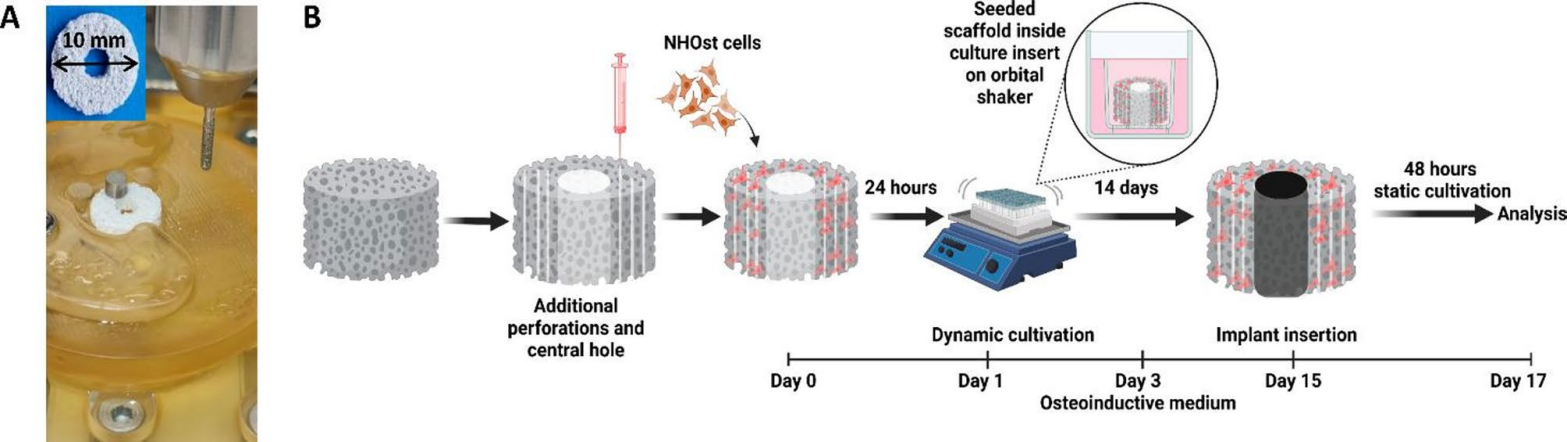
Experimental design and HA/TCP scaffold adjustment to cultivate the 3D in vitro bone-implant model. (**A**) Drill guide used for preparation of implant insertion area using a 2.5 mm round end taper dental bar. (**B**) Schematic illustration of experimental setting including timeline (created with BioRender.com)

Thus, prior to seeding of NHOst cells, 5–6 perforations to open and connect closed cavities within the scaffold material were applied using a syringe. Moreover, an in-house designed and manufactured drill guide for 2.5 mm round end taper dental bar was applied to enable a highly precise and reproducible placement of a gap area within the spongy but brittle material for subsequent insertion of the implant (Fig. 2A). During cultivation, the cell-loaded scaffolds were subjected to orbital shaking to improve nutrient, metabolite and oxygen supply. With these modifications, an optimized protocol for the assembly and analysis of 3D in vitro bone-implant models could be established (Fig. 2B).

### Osteoblast viability and growth in the bone-implant model

**Fig. 3:**
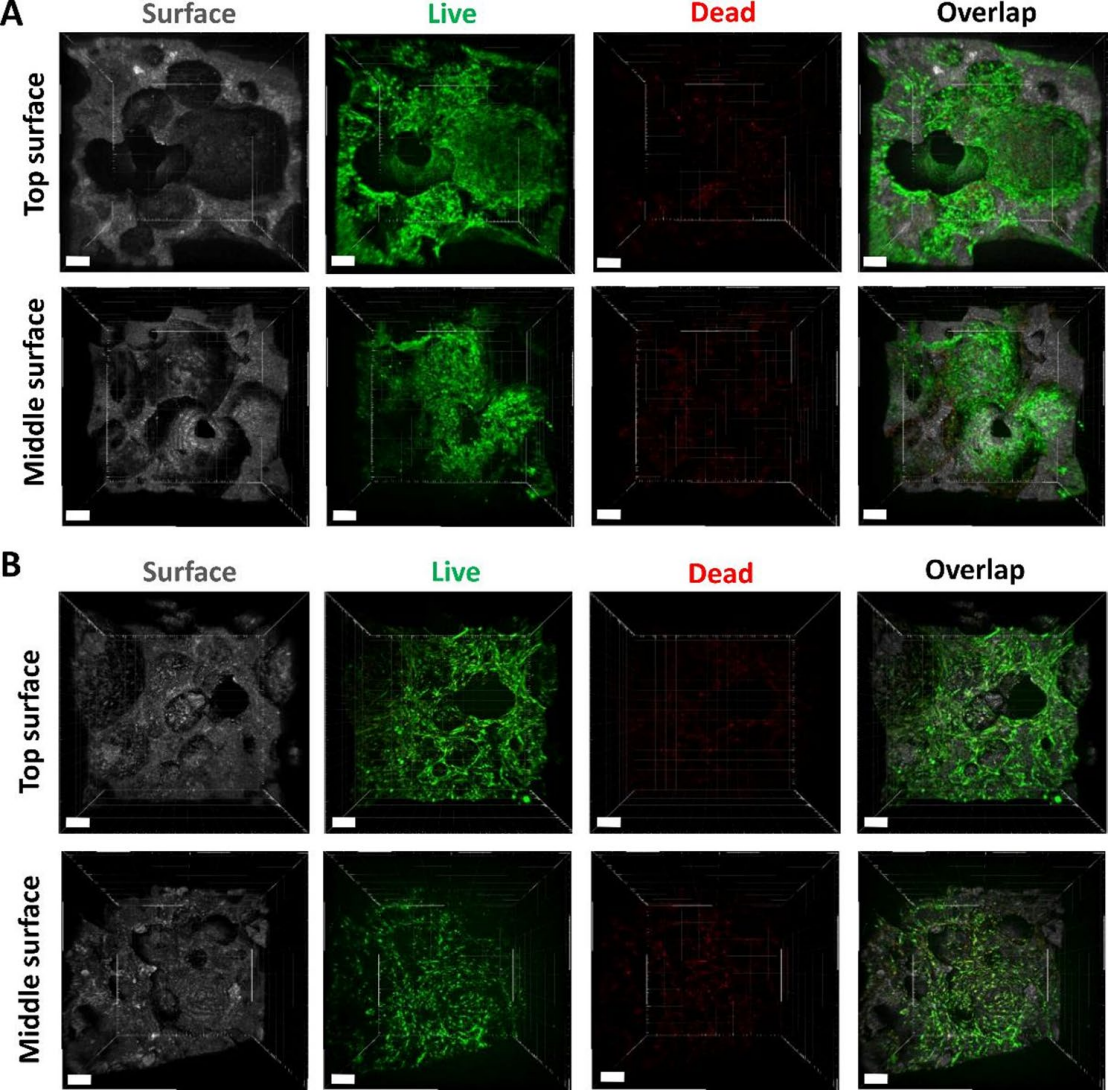
Cell growth and viability on scaffolds (during 3D in vitro bone-implant model development). (**A**, **B**) Live/dead images of NHOst cells on HA/TCP scaffolds at day 1 (**A**) and day 17 (**B**) after seeding. Images were taken on the top of the scaffold and in the middle by cutting the scaffolds perpendicularly. Scaffold surface was visualized using reflection in CLSM. Live cells, green; Dead cells, red; scaffold surface, grey. Data shown are representative of *n* = 6 independent experiments. Scale bars represent 200 μm

Using the optimized protocol specified above, live/dead staining was used to determine the cell viability and growth. One day after seeding, cells exhibited good viability and elongated morphology on the surface as well as in the middle of the scaffold (Fig. 3A). Cells exhibited extreme high density and uniform distribution only on the scaffold’s surface, while moderate cell densities were observed deeper within the scaffold material. However, a considerable fraction of cells were able to advance throughout the scaffold, particularly in interconnected cavities with direct access to the surface (Fig. 3A). After 17 days of dynamic cultivation, the cells on top as well as within the material displayed predominantly green fluorescence, indicating high cell viability and robust growth (Fig. 3B). Although red fluorescence was slightly increased in the middle, suggesting less optimal conditions for cell survival, the overall high cell viability confirmed sufficient supply even over longer culture periods (Fig. 3B). Importantly even after 23 days of cultivation no loss of viability was observed (Fig. S2). These findings confirm that the scaffold and cultivation method provided a supportive environment for cell survival and proliferation in both regions, with strong viability even in the middle of scaffold.

### Expression of osteoblastic phenotype in the bone-implant model

**Fig. 4:**
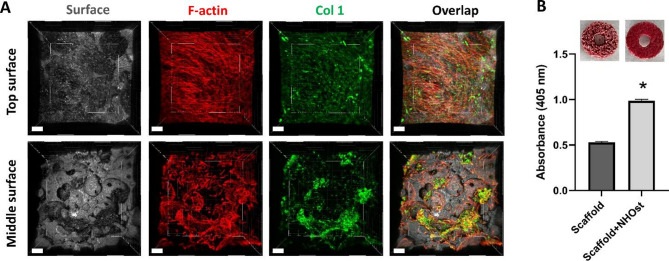
Evaluation of osteoblastic phenotype on seeded scaffolds after 17 days of cultivation in 3D in vitro bone-implant model. (**A**) Immunofluorescence images for collagen type 1 formation and F-actin (representation of the cell cytoskeleton). Col 1, green; F-actin, red; scaffold surface, grey. Images were taken on the top of the scaffold and in the middle by cutting the scaffolds perpendicularly. Scaffold surface was visualized using reflection in CLSM. Data shown are representative of *n* = 4 independent experiments. Scale bars represent 150 μm. (**B**) Quantification of Alizarin Red S Staining in scaffolds without cells and seeded scaffolds with normal human osteoblasts after 17 days cultivation. Data shown are representative of *n* = 3 independent experiments. Values are presented as mean ± SD. Stars indicate a statistically significant increase compared to the control (scaffold without cells) with *p* ≤ 0.05

To assess the capacity of osteoblasts in the 3D model to form extracellular matrix, collagen type 1 immunofluorescence staining was performed. As depicted in Fig. 4, expression of collagen type 1 was confirmed throughout the 3D bone-implant model. While collagen distribution in the middle of the scaffold was not as uniform as on the surface, this variation is expected due to the accumulation of cells mainly around pathways with direct access to the surface (Fig. 4A). Cell cytoskeleton stained with phalloidin showed an intact elongated morphology of cells after 17 and 23 days of osteogenic cultivation on both surface and in the middle of the scaffold (Fig. 4A and Fig. S3). ARS staining highlighted also an increased formation of mineralized ECM over 17 days of cultivation (Fig. 4B). Therefore, these findings confirmed the sustained expression of phenotypic characteristics of osteoblasts with ECM mineralization and collagen expression in HA/TCP scaffolds under the applied conditions.

### Assembly and histological characterization of the peri-implant bone-mucosa model

**Fig. 5:**
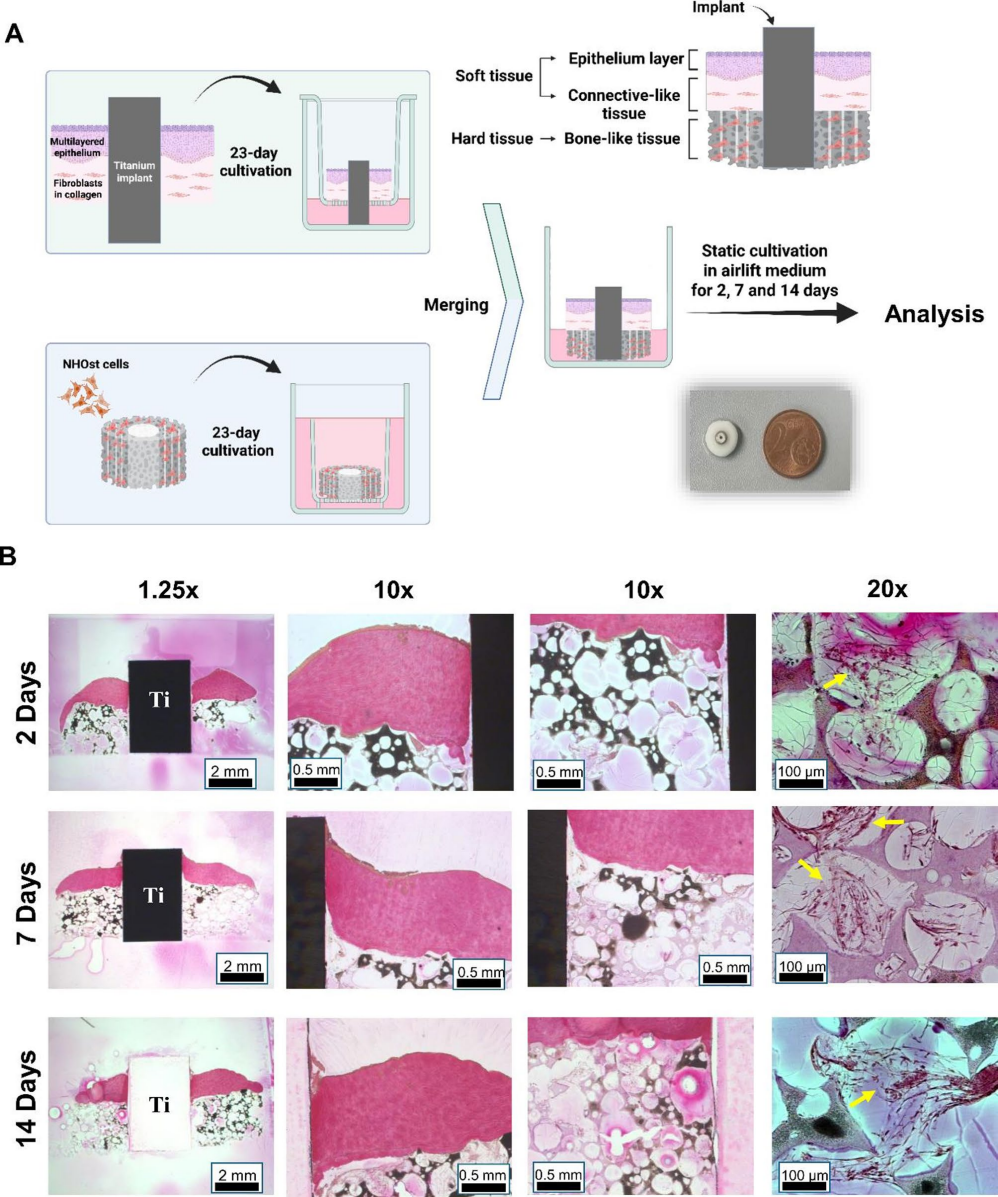
Experimental design and histological sections of 3D in vitro peri-implant bone-mucosa composite model. (**A**) Schematic illustration of experimental setting for 3D composite model (created with BioRender.com). (**B**) Van Gieson stained histological sections of the 3D composite model at 2, 7, and 14 days of static cultivation following the merging of soft and hard tissue. 1.25× = overview of complete model including soft tissue, implant and hard tissue; 10× = soft tissue, implant, hard tissue and their interfaces in higher magnification; 20× = hard tissue region. Arrows indicate the osteoblasts imbedded in secreted matrices. Data shown are representative of *n* = 3 independent experiments

The different steps of assembling the peri-implant bone-mucosa model, along with a schematic illustration and a photograph of the final product, are shown in Fig. 5A. Histological staining and analyses at all time points demonstrated a composite structure including oral mucosa tightly attached to an underlying hard tissue (Fig. 5B). The top layer exhibited a stratified epithelium covering collagen-embedded human gingival fibroblasts. Within 14 days of airlift cultivation this epithelium remained intact without any obvious indication of degradation or separation. The bone-mucosa interface appeared closely connected without bigger gaps or invading cells. In fact, the soft tissue even followed the topographical outline of the osteoblast-loaded scaffold indicating no rejection of the different materials and cells. In addition, the interfaces of the implant with both, soft- and hard-tissue, demonstrated no signs of invading cells (especially no apical migration of epithelium), which confirmed the sustained interaction especially of the hydrogel-imbedded fibroblasts with the implant. The pores of the scaffolds, contained osteoblasts imbedded in secreted matrices in variable amounts (Fig. 5B). Overall, these findings demonstrate the successful assembly of the 3D peri-implant bone-mucosa model, closely resembling the oral tissue-implant structure with the predominant cell types.

### Osteoblast viability and growth in the peri-implant bone-mucosa model

**Fig. 6:**
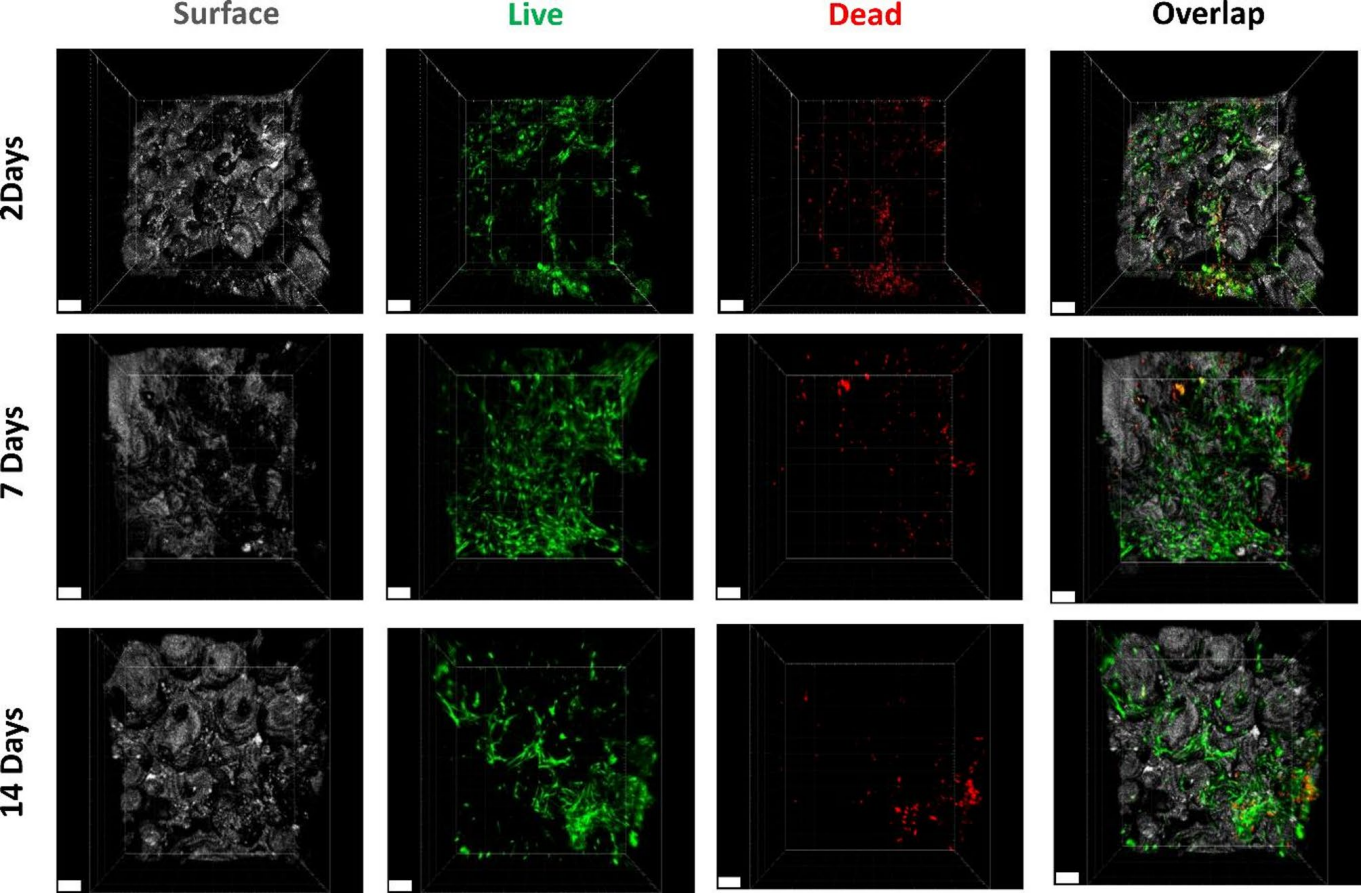
Cell growth and viability of NHOst cells on scaffolds at 2, 7 and 14 days of static cultivation in 3D in vitro peri-implant bone-mucosa composite model. Images were taken from the middle of the scaffold by cutting the composite model perpendicularly. Scaffold surface was visualized using reflection in CLSM. Live cells, green; Dead cells, red; scaffold surface, grey. Data shown are representative of *n* = 3 independent experiments. Scale bars represent 100 μm

Osteoblast cell viability and growth within the peri-implant bone-mucosa model was assessed using live/dead staining after merging artificial mucosa and bone model for 2, 7 and 14 days. Due to the close connection of the bone-mucosa interface they could not be separated without damage. Accordingly, a simple microscopic assessment of cells on the top was no longer possible, but was limited to the state of cells in the middle of the scaffold. Cells demonstrated high density and viability even within the hard tissue indicating no adverse effects of combination with oral mucosa and cultivation in air-lift medium at any time point. (Fig. 6).

### Expression of osteoblastic phenotype in the peri-implant bone-mucosa model

**Fig. 7:**
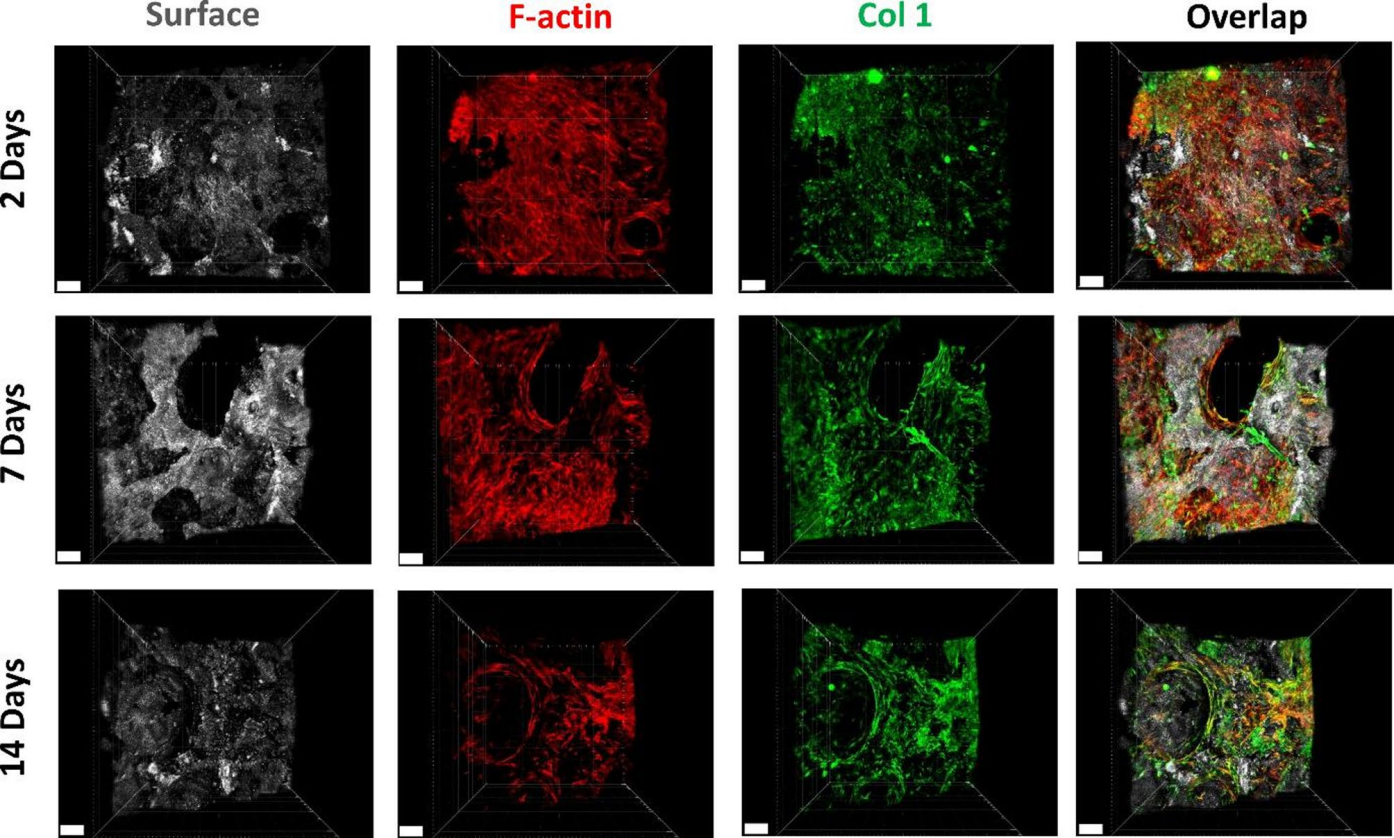
Evaluation of osteoblastic phenotype on seeded scaffolds at 2, 7 and 14 days of static cultivation in 3D in vitro peri-implant bone-mucosa composite model. Immunofluorescence images for collagen type 1 formation and cell cytoskeleton. Col 1, green; F-actin, red; scaffold surface, grey. Images were taken from middle of scaffold by cutting the composite model perpendicularly. Scaffold surface was visualized using reflection in CLSM. Data shown are representative of *n* = 3 independent experiments. Scale bars represent 100 μm

After 2, 7 and 14 days of cultivation in the 3D composite model, immunofluorescence staining confirmed the continued expression of collagen type 1 in the hard tissue (Fig. 7). The distribution of collagen was comparable to the pattern observed in the 3D bone-implant model alone. Phalloidin staining demonstrated the spread of cells within the scaffold (Fig. 7). Compared to the 3D bone-implant model alone, the presence of the oral mucosa or the use of air-lift medium did not interfere with collagen expression in underlying hard tissue.

### Multispecies biofilm (MSBF) integration in the peri-implant bone-mucosa model

**Fig. 8:**
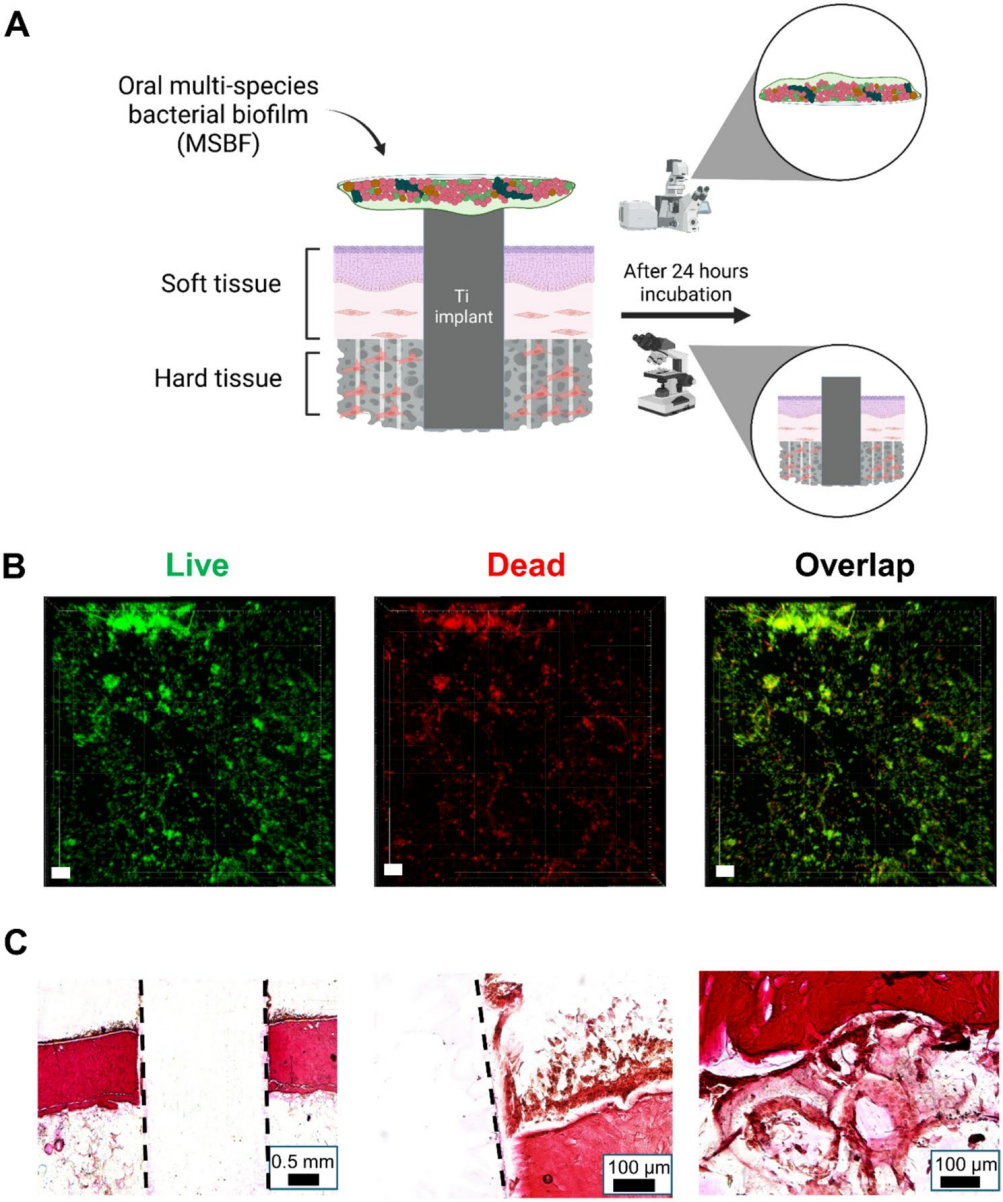
Integration and characterization of MSBF in co-culture model of 3D in vitro peri-implant bone-mucosa-biofilm composite model. (**A**) Schematic illustration of co-culture model with integration of MSBF (created with BioRender.com). (**B**) Representative 3D-reconstructed CLSM images of biofilms after 24 h co-cultivation with a 14-day-old 3D composite model. Viable bacteria are shown in green and dead bacteria in red. Data shown are representative of *n* = 1 experiment. Scale bars represent 10 μm. (**C**) Van Gieson stained histological sections of 3D composite model following co-cultivation with MSBF for 24 h; Left (2.5x) = overview; middle (20x) = mucosa part and mucosa/implant interface; right (20x) = bone/mucosa interface. Dashed lines show the implant-tissue border

As an outlook experiment, a MSBF was incorporated into the 3D composite model to create a co-culture system based on a 3D in vitro peri-implant bone-mucosa-biofilm model, as illustrated in the schematic in Fig. 8A. Live/dead staining of the biofilms confirmed the successful integration of a vital biofilm into the 3D composite model using conditions established in previous studies for co-cultivation of 3D models and bacterial biofilms [20, 25](Fig. 8B). The histological images demonstrate a continued tissue connection to implant surface, even after 24 h of co-cultivation with MSBF (Fig. 8C). The epithelium was damaged and loosened showing early signs of degradation, particularly near the embedded implant. The bone–mucosa interface exhibited intact and continuous contact (Fig. 8C).

## Discussion

In the oral cavity, dental implants interact with multiple interconnected biological structures including oral mucosa, underlying bone and the surrounding microbiome. These elements influence each other contributing to peri-implant health and playing a critical role in the development of peri-implant diseases [7, 8, 28]. Studying these conditions or developing strategies to mitigate implant-related complications by focusing on only one factor—be it soft tissue, bone, or bacteria—without considering their interplay fails to capture the complexity of the clinical environment. In a similar way, relying on conventional 2D in vitro models Limits the clinical significance of findings since the behavior and dynamic interaction of cells is different in 3D tissue structures [15, 22]. Consequently, there is a growing demand for advanced peri-implant models that closely replicate the clinical tissue-implant interface [21]. Therefore, the aims of the present study were (i) to develop a 3D in vitro bone-implant model and (ii) to combine this model with our previously developed 3D peri-implant mucosa model to create the most complex modular 3D in vitro peri-implant bone-mucosa composite model that accurately represents relevant factors in vivo. This complex 3D composite model will provide a clinically relevant platform for studying tissue-implant interactions, evaluating modified implant surfaces/biomaterials and investigating early peri-implant disease development. However, depending on the research interest, major cell types relevant for investigation of progressed infection and inflammation, Like immune cells, are still missing and are subject to continued development of our 3D model system. To study peri-implant infections at all, the combination and co-cultivation of the new 3D model with oral biofilms is necessary. Therefore, we addressed the possible integration of a multispecies biofilm in the model for further applications.

In order to study the osseointegration processes and implant material properties in vitro, different strategies have been followed, including a tissue-on-chip model [29], wrapping of cell-sheets around implant [30], explant models [31–33] and most commonly, scaffold-based models relying on materials such as hydrogels or bone blocks as scaffolds [21, 34–37]. 3D culture systems using scaffolds offer an animal-independent approach that enables controlled, time-resolved investigation of disease development [21]. Based on the published findings and applications of different scaffold materials, we focused on few promising candidates which are commercially available for our purpose [15, 38–40]. PCL polymer has been widely utilized as a scaffold material in tissue engineering applications due to its excellent biocompatibility, low immunogenicity and optimal degradation properties [40]. Likewise, PS scaffolds, characterized by their high stiffness and non-resorbable properties, have been used as bone substitutes and supporting structures for 3D in vitro cultivation [38, 39]. HA/TCP, composed of hydroxyapatite and calcium phosphate, closely resembles the chemical composition of natural bone and is already used clinically as a bone graft substitute. Additionally, their macroscopic trabecular architecture resembles the spongy structure of cancellous bone [41–43]. Tested scaffolds used in this study—PS, PCL, and HA/TCP— demonstrated good cell adhesion in quantity, quality and viability, with HA/TCP scaffolds exhibiting the highest cell seeding efficiency (CSE). However, after prolonged cultivation in osteogenic medium, cells on PS and PCL scaffolds did not further distribute over the scaffold volume and remained mainly in the locations defined by the drop-seeding. In contrast, HA/TCP scaffolds exhibited superior cell distribution, growth and osteoblastic phenotype likely due to their calcium-based composition, which imparts osteoinductive properties [43]. Considering the high resemblance of natural bone structure in terms of chemical and physical properties by the HA/TCP scaffold, it was preferred for the incorporation in 3D model assembly. To improve cell penetration and viability in the center of HA/TCP scaffolds, modifications were implemented including pre-wetting scaffolds to remove air bubbles, adding a central implant hole with perforations, and reducing scaffold thickness. A similar HA/TCP scaffold was used in a previous study with 3 mm thickness to develop an engineered bone model, although cellular vitality decreased after three months of cultivation [44]. In our study, we used a 2.5 mm thick scaffold and a shorter cultivation period, which helped maintain better cellular vitality. Additionally, static cultivation can be inefficient to support cell growth beside the periphery of scaffolds due to Limited diffusion of essential nutrients and oxygen, with maximum cell ingrowth within a range of 200–400 μm from the outer surface of a scaffold [45, 46]. Simple dynamic cultivation methods can improve cell growth and expression of osteoblastic phenotype [47]. Therefore, dynamic cultivation on an orbital shaker was implemented together with inserts and perforated membranes to enhance the flow of medium through scaffolds. After optimizing scaffold adjustment, cultivation system and seeding technique, our results demonstrated sustained cell viability and growth throughout the scaffold thickness over extended periods, highlighting the positive effects of the adjusted protocol. The presented model is in line with the results of other studies evaluating osteoblast viability on HA scaffolds and attesting its good biocompatibility [48–50]. Moreover, by using primary human osteoblasts, which more accurately reflect the physiological properties of natural tissue cells [43], the clinical relevance of this model could be increased, although a persistent osteogenic differentiation in the merged model could not be demonstrated so far.

Previous investigations have demonstrated that primary osteoblasts cultured in osteogenic medium within a 3D cultivation setup exhibit collagen type I (Col 1) expression, a marker of early-stage maturation, by day 7 with mineralization occurring by day 14 [51]. Therefore, we selected a 17-day cultivation period, with 14 days dedicated to osteoblastic maturation, similar to Almela et al. [50]. In contrast, here a titanium cylinder representing a dental implant was inserted into the model and cultivated for the last two days, creating a standalone bone-implant model. This approach aimed to maintain simplicity while providing a more clinically relevant alternative to 2D cultivation methods simulating the immediate post-implant placement conditions in the oral cavity. As a consequence of cell viability, the cultivation period could be extended to 23 days, allowing for further tissue maturation before transitioning to airlift conditions and integrating with the mucosa model. Evaluation of resulting bone-like tissue formation revealed key features characteristic for extracellular matrix (ECM) of bone, including the presence of Col 1 and enhanced mineralization. Bone ECM is made up by organic (90% composed of Col 1 and 10% non-collagenous proteins) and inorganic compounds (hydroxyapatite). It modulates cell adhesion, proliferation, differentiation, bone strength and functionality of mature bone [52]. The active production of ECM components within our 3D setting reflects the expression of osteoblastic phenotype, as it was demonstrated also in other studies using a variety of cell types and scaffold materials [21, 37, 43, 53]. Overall, our results confirm the successful development of a clinically relevant, viable 3D bone-implant model with robust ECM formation and mineralization, establishing hard tissue for the subsequent integration within the anticipated 3D composite model. However, in the version presented here, the model does not yet take into account the complex topography of dental implants and long-term cultivation after implant insertion, which would be necessary to represent progressed osseointegration.

A recent systematic review of available in vitro 3D complex models in implant dentistry highlighted that typically the implant interface behavior with either bone or gingival tissue was addressed in these models, but not both [21]. In the present study, we addressed this limitation by merging our 3D bone model with our previously developed oral mucosa model with an integrated titanium implant [25]. This created the first and most complex model of oral tissue, consisting of hard and soft tissue, surrounding a dental implant. Histological evaluations revealed structures with bone tissue-like, connective tissue-like and epithelium layers on top of each other arranged around the titanium implant, closely resembling the natural architecture of peri-implant tissues within the oral cavity. Similar histological observations have been reported in previous studies that developed comparable bone-oral mucosal models but without simultaneous integration of an implant [44, 50]. Moreover, unlike previous investigations which employed a fibrin-based adhesive sealant to permanently merge hard and soft tissues [44, 50], our model does not rely on the additional use of an adhesive sealant. Although fibrin-based adhesive sealants can effectively hold tissues together and close gaps, they may also act as a mechanical barrier, impairing the direct interactions between tissues [54]. Such interactions are based on physical contact within and between soft and hard tissues as well as the exchange of signaling molecules, secreted growth factors and enzymes, but also inflammatory cytokines in response to bacterial infections [55, 56]. Our focus for the future application of the composite model presented here will be on the investigation of early manifestation of peri-implant diseases (shift from homeostasis to dysbiosis) and the evaluation of possible preventive and therapeutic strategies. Therefore, it was essential that the integrity of the sterile tissue structures could be maintained over a longer period of time. This primarily affects the differentiation of the epithelium, which can only be maintained in airlift with appropriate media. While similar studies have successfully cultivated bone-oral mucosal constructs for 5 days in an air/liquid interface [44, 50], we were able to extent this period to 14 days, without losing epithelial integrity, despite limited access to medium from below through the scaffold. In addition, a strong implant-mucosa bond plays a crucial role in mimicking a healthy peri-implant situation. In this regard, no mucosal detachment from the titanium or increased migration of epithelial cells was observed during the study period, likely due to the separate mucosa formation with an integrated implant [25], and then careful transfer onto the pre-populated hard tissue scaffold using an extended implant serving as a stabilizing connection. Moreover, osteoblasts cell viability and osteoblastic phenotype was maintained within the peri-implant bone-mucosa model, consistent with previous studies [44, 50]. Consequently, we were able to produce a controlled 3D environment encompassing multiple cell types which can not only coexist but have the possibility to interact dynamically, exchanging signals and influencing each other’s behavior — closely mimicking the physiological interplay at the implant interface.

Peri-implant diseases are complex multifactorial conditions involving both soft tissue and hard tissues arising from a disrupted host-microbe balance [7]. Various organotypic oral tissue models, including epithelium and gingiva/mucosa, have been integrated with bacterial, fungal, or viral species to study host-microbe dynamics [22]. Some studies have also incorporated titanium implants into mucosa model to assess host-microbe interactions at peri-implant sites [20, 23, 25, 57]. In this study, we exemplary integrated an oral multispecies biofilm (MSBF) into our 3D peri-implant bone-mucosa model to demonstrate the potential for simulating host-material-microbe interactions in a controlled environment, providing a robust platform for future research. This biofilm comprises four oral bacterial species, including early, middle and late colonizers, representing an early commensal biofilm on the dental implant surface, closely resembling clinical situations [27]. Notably, the biofilm remained viable after integration with the peri-implant bone-mucosa model, demonstrating its ability to sustain activity within the model and further enhancing its clinical relevance for studying the dynamic interactions between host tissues and microbial communities. Furthermore, the epithelial barrier was compromised and detached from the underlying tissue, a hallmark of peri-implant diseases, indicating disruption of the host-microbe homeostasis [20, 58]. This may be attributed to the downregulation of adhesion-related genes, as reported in a previous study [20]. However, a notable distinction here is that the epithelium damage was already observed after 24 h of co-cultivation, whereas Mikolai et al. demonstrated a protective pro-inflammatory response in the peri-implant mucosa model and a compromised host microbe balance not before 48 h [20]. This difference may be attributed to the presence of osteoblasts, which are known to play a role in immune modulation and inflammatory response to bacterial infection. Previous studies have demonstrated that osteoblasts co-cultured with bacteria actively secrete pro-inflammatory cytokines and chemokines [59–61]. Therefore, their presence in our model may have influenced cytokine signaling, potentially accelerating the disruption of epithelial integrity, which should be addressed in subsequent studies. The mutual influence of different tissues and bacterially induced infections in complex models could also contribute to the in-depth investigation of further relationships. Pro-inflammatory cytokines such as interleukin-1β, interleukin-6, and tumor necrosis factor can impair osteogenic differentiation and promote osteoclast activation, leading to bone resorption in peri-implantitis [62, 63]. Other factors with high relevance for the development and progression of peri-implant infections are different implant materials and surface characteristics [64] but also inclusion of immune cells [65] or the consideration of different patient backgrounds (like gender, age or pre-conditions), which will be considered in future studies and further developments of our model. Further efforts would also need to be made to enable more in-depth investigation of advanced inflammatory processes, including bone loss during peri-implantitis, and relevant aspects such as mechanical stress and foreign body reactions in the 3D model [66, 67]. The recent version of the 3D peri-implant bone-mucosa model offers the possibility of evaluating pro-inflammatory responses to biofilm infections in a system that, for the first time, considers both soft and hard tissue. The model represents the peri-implant situation shortly after implantation and provides valuable insights into the function and mutual influence of various tissue cells in both healthy and diseased states. Further studies are planned to use the model for detailed investigations in the field of early host-biofilm interactions, as well as the development of innovative approaches in diagnostics, prevention and therapy.

## Conclusion

The present study successfully established a 3D bone-implant model by seeding and differentiating primary osteoblasts on a HA/TCP scaffold, which closely mimics the chemical and physical properties of natural bone. This scaffold-based approach provides a more clinically relevant alternative to traditional 2D models. The integration of this bone model in a 3D peri-implant mucosa model led to the development of the first and most complex 3D in vitro peri-implant model to date. This 3D peri-implant bone-mucosa model successfully replicates key elements of the clinical setting, including soft tissue, hard tissue, and an embedded dental implant. Notably, the insertion of the implant after bone-like tissue formation further validated its resemblance to clinical setups. Besides, the incorporation of MSBF presents a promising step toward studying early pro-inflammatory responses to bacterial infections within a physiologically relevant peri-implant environment. Consequently, the new developed 3D peri-implant bone-mucosa model will enable, on the one hand, future investigations of the host-biofilm interactions to provide new knowledge about peri-implant diseases. On the other hand, it will allow the testing of new preventive and therapeutic strategies in a clinically relevant 3D in vitro setting.

## Supplementary Information

Supplementary Material 1.

## Data Availability

All data generated or analyzed during this study are included in this published article [and its supplementary information files].

## Abbreviations

2D/3D: Two/ Three-dimensional
MSBF: Multispecies biofilm
CLSM: Confocal Laser Scanning Microscopy
PS: Polystyrene
PCL: Polycaprolactone
HA/TCP: Hydroxyapatite, β-Tricalcium phosphate
ARS: Alizarin Red S Staining
NHOst: Normal Human Osteoblasts
HGF: Human Gingival Fibroblasts
OKF6: Immortalized Human Oral Keratinocytes
Col 1: Collagen type-I
Ti: Titanium
FBS: Fetal Bovine Serum
P/S: Penicillin/Streptomycin
KerSFM: Keratinocyte Serum-Free Medium
BPE: Bovine Pituitary Extract
EGF: Epithelial Growth Factor
CSE: Cell Seeding Efficiency
BHI: Brain Heart Infusion
PFA: Paraformaldehyde
ECM: Extracellular Matrix
