# Supplementary material for: Establishment of a three-dimensional in vitro peri-implant bone-mucosa composite model
Source: BMC Oral Health. 2026 Jan 14;26:91. doi: 10.1186/s12903-025-06930-2 (PMC12801871; doi:10.1186/s12903-025-06930-2)
Supplement: Supplementary file 1 — Supplementary Material 1. [file 12903_2025_6930_MOESM1_ESM.docx]

**Establishment of a three-dimensional *in vitro* peri-implant bone-mucosa composite model**

Behnaz Malekahmadi ^1,2 #^, Marjan Kheirmand-Parizi ^1,2 #^, Carina Mikolai ^1,2 *^, Andreas Winkel ^1,2^ , Muhammad Imran Rahim ^1,2^, Katharina Doll-Nikutta ^1,2^, Andreas Kampmann ^3^, Nils-Claudius Gellrich ^3^, Dagmar Wirth ^4,5^, Henning Menzel ^6^, Meike Stiesch^1,2 *^

1. Department of Prosthetic Dentistry and Biomedical Materials Science, Hannover Medical School, Carl-Neuberg-Str. 1, 30625 Hannover, Germany.
2. Lower Saxony Centre for Biomedical Engineering, Implant Research and Development (NIFE), Stadtfelddamm 34, 30625 Hannover, Germany.
3. Department of Oral and Maxillofacial Surgery, Hannover Medical School, Carl-Neuberg-Strasse 1, 30625 Hannover, Germany.
4. Model Systems for Infection and Immunity, Helmholtz Centre for Infection Research, Inhoffenstraße 7, 38124 Braunschweig, Germany.
5. Institute of Experimental Medicine, Hannover Medical School, Carl-Neuberg-Str. 1, 30625 Hannover, Germany.
6. Institute for Technical Chemistry, Braunschweig University of Technology, Hagenring 30, 38106 Braunschweig, Germany.

# These authors contributed equally to this work.

Corresponding authors: Mikolai.Carina@mh-hannover.de, Stiesch.Meike@mh-hannover.de

**Supplementary Information**

**
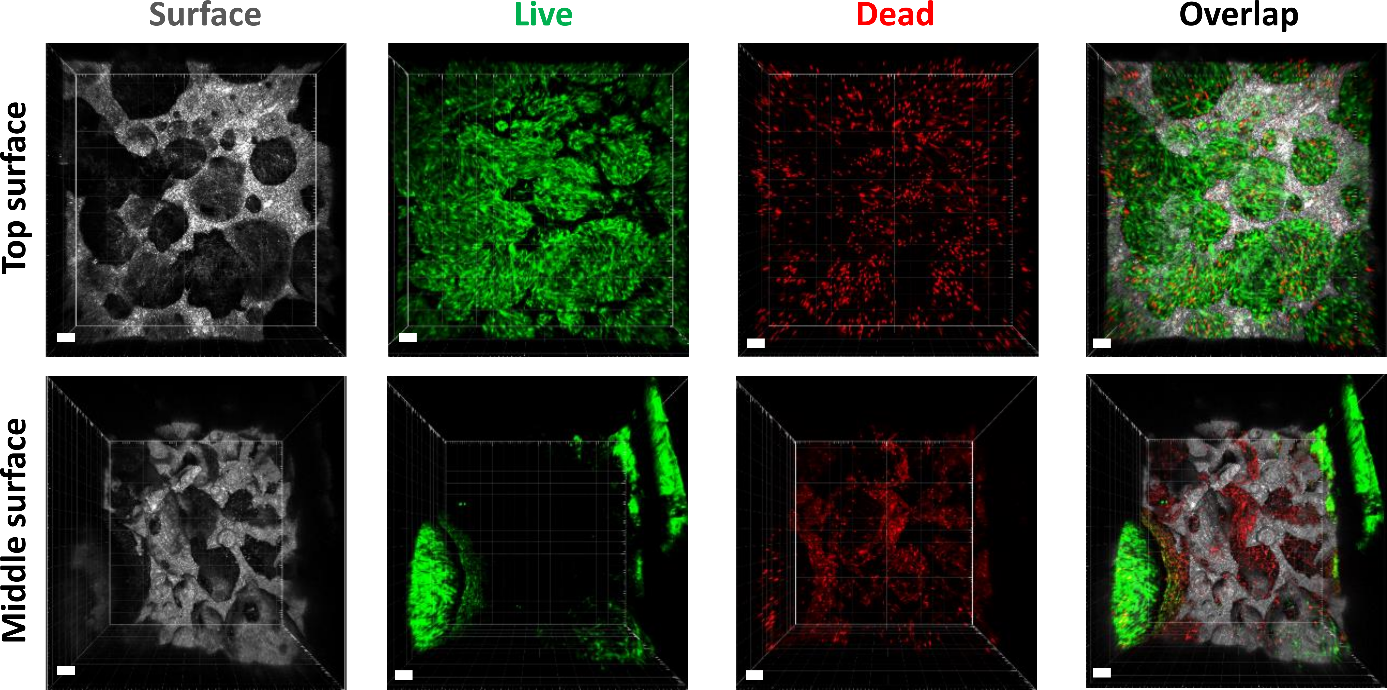
**

**Figure S1. Live/dead images of NHOst cells on 10×2mm HA/TCP scaffolds (without holes) after 17 days cultivation.** Data shown are representative of n=2 independent experiments. Scale bars on the top surface represent 150 µm and those on the middle surface represent 200 µm.

**
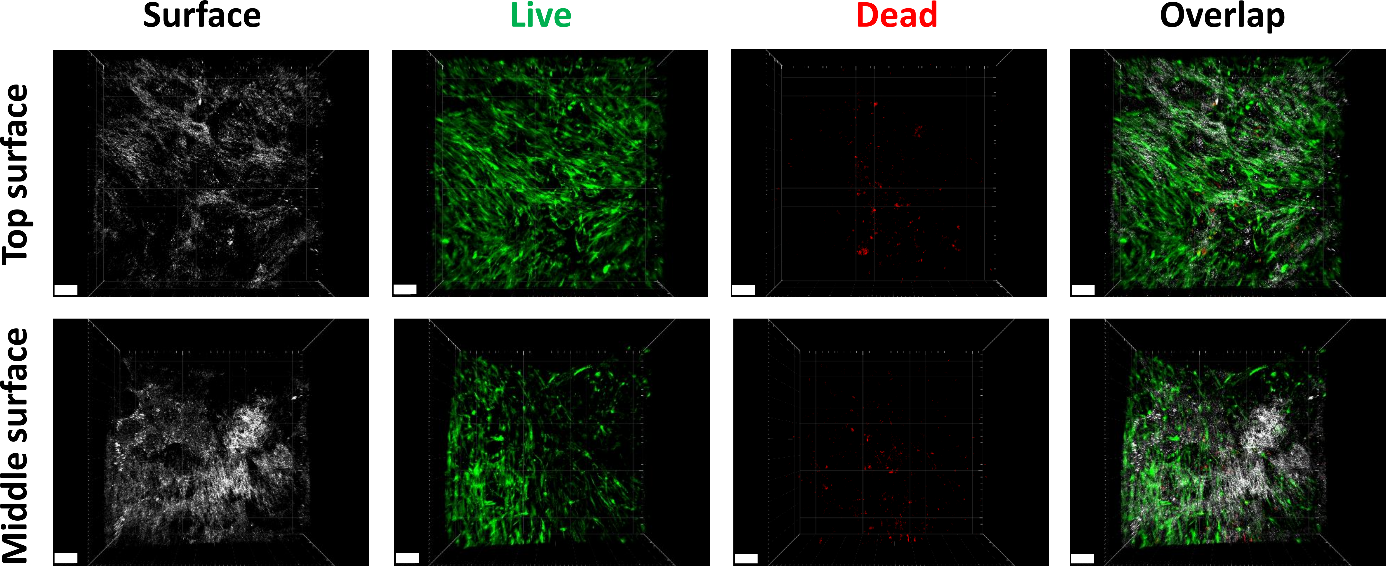
**

**Figure S2. Cell growth and viability on scaffolds after 23 days of cultivation in 3D in vitro bone model.** Live/dead images of NHOst cells on HA/TCP scaffolds at day 23 after seeding. Images were taken on the top of the scaffold and in the middle by cutting the scaffolds perpendicularly. Scaffold surface was visualized using reflection in CLSM. Live cells, green; Dead cells, red; scaffold surface, grey. Data shown are representative of n=4 samples from a single experiment. Scale bars represent 100 µm.


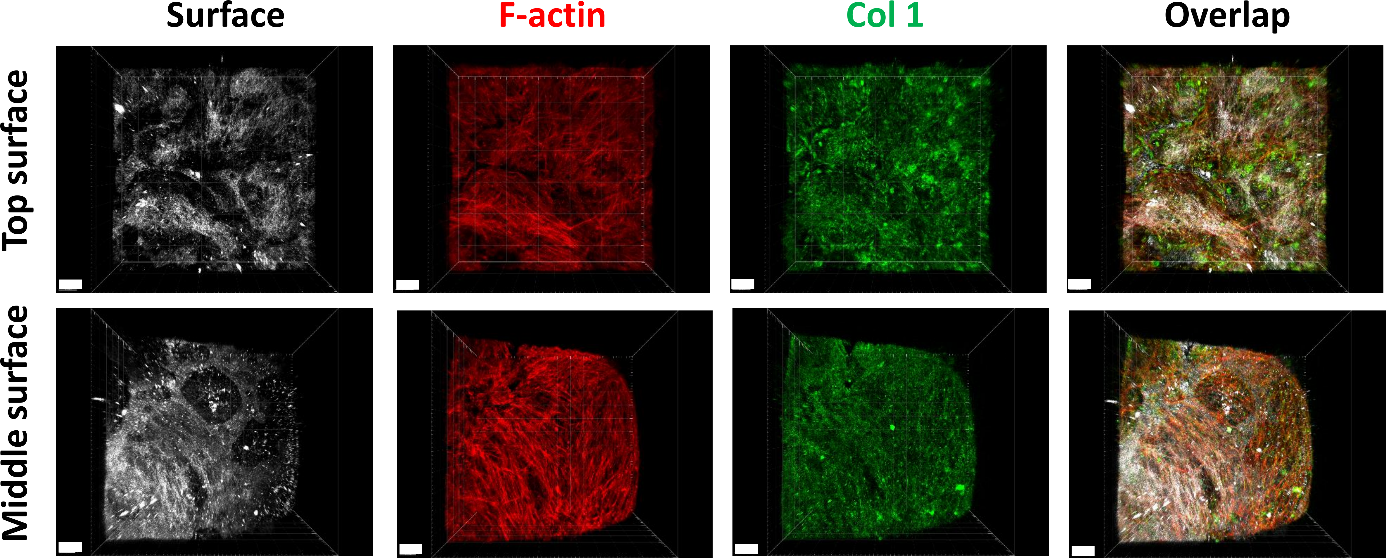


**Figure S3. Evaluation of osteoblastic phenotype on seeded scaffolds after 23 days of cultivation in 3D in vitro bone model.** Immunofluorescence images for collagen type 1 formation and cell cytoskeleton. Col 1, green; F-actin, red; scaffold surface, grey. Images were taken on the top of the scaffold and in the middle by cutting the scaffolds perpendicularly. Scaffold surface was visualized using reflection in CLSM. Data shown are representative of n=4 samples from a single experiment. Scale bars represent 100 µm.
